# Effects on Contralateral Muscles after Unilateral Electrical Muscle Stimulation and Exercise

**DOI:** 10.1371/journal.pone.0052230

**Published:** 2012-12-20

**Authors:** Yafeng Song, Sture Forsgren, Jiguo Yu, Ronny Lorentzon, Per S. Stål

**Affiliations:** 1 Department of Integrative Medical Biology, Section for Anatomy, Umeå University, Sweden; 2 Department of Surgical and Perioperative Sciences, Sports Medicine Unit, Umeå University, Umeå, Sweden; Brigham and Women’s Hospital, Harvard Medical School, United States of America

## Abstract

It is well established that unilateral exercise can produce contralateral effects. However, it is unclear whether unilateral exercise that leads to muscle injury and inflammation also affects the homologous contralateral muscles. To test the hypothesis that unilateral muscle injury causes contralateral muscle changes, an experimental rabbit model with unilateral muscle overuse caused by a combination of electrical muscle stimulation and exercise (EMS/E) was used. The soleus and gastrocnemius muscles of both exercised and non-exercised legs were analyzed with enzyme- and immunohistochemical methods after 1, 3 and 6 weeks of repeated EMS/E. After 1 w of unilateral EMS/E there were structural muscle changes such as increased variability in fiber size, fiber splitting, internal myonuclei, necrotic fibers, expression of developmental MyHCs, fibrosis and inflammation in the exercised soleus muscle. Only limited changes were found in the exercised gastrocnemius muscle and in both non-exercised contralateral muscles. After 3 w of EMS/E, muscle fiber changes, presence of developmental MyHCs, inflammation, fibrosis and affections of nerve axons and AChE production were observed bilaterally in both the soleus and gastrocnemius muscles. At 6 w of EMS/E, the severity of these changes significantly increased in the soleus muscles and infiltration of fat was observed bilaterally in both the soleus and the gastrocnemius muscles. The affections of the muscles were in all three experimental groups restricted to focal regions of the muscle samples. We conclude that repetitive unilateral muscle overuse caused by EMS/E overtime leads to both degenerative and regenerative tissue changes and myositis not only in the exercised muscles, but also in the homologous non-exercised muscles of the contralateral leg. Although the mechanism behind the contralateral changes is unclear, we suggest that the nervous system is involved in the cross-transfer effects.

## Introduction

There is a wide range of examples in the literature showing that unilateral intervention produces bilateral effects. The measured effects vary from adaptations of muscle performance to alterations in gene expression, inflammation and tissue remodeling [1]. Previous studies have shown that unilateral strength training can increase the strength not only in the trained muscle but also in the homologous muscle of the contralateral limb [2], [3], [4]. This cross-transfer effect has been reported both for small and large limb muscles and in response to different types of exercises as well as after unilateral electrical muscle stimulation (EMS) [5], [6], [7], [8]. However, there are also reports that deleterious events can be cross-transferred as well. There is thus a strictly symmetrical distribution of inflammation in some chronic inflammatory diseases [9], [10] and a presence of a mirror image of the nervous system for pain [11], [12]. Furthermore, painful Achilles tendinopathy frequently develops bilaterally after muscle/tendon overuse [13], [14] and recent findings indicate that there are bilateral cross-transfer effects after unilateral treatment [15]. Although the mechanism for the contralateral increase in strength after unilateral exercise may differ from the symmetrical distribution in inflammation and pain and the bilateral effects after unilateral tendon treatment, the effects have been proposed to be mediated through the central nervous system [1], [9], [10], [11], [12], [16], [17], [18], [19], [20]. The previous findings that peripheral nerve lesions can affect contralateral non-lesioned neurons, further support cross-transfer signaling through the nervous system [1].

When properly performed, exercise can provide significant functional benefits that includes increased muscle mass, strength and resistance to fatigue. However, pronounced muscle overuse, and especially that due to unaccustomed eccentric exercise and electrical stimulation, can also induce muscle damage. This injury is generally characterized by changes in fiber morphology and an increased amount of connective tissue, as well as muscle fiber degeneration, necrosis and inflammation [21], [22], [23], [24], [25], [26], [27], [28]. To date, there is no information available that clarifies if unilateral muscle overuse leading to muscle damage is accompanied by morphological alterations and inflammation (myositis) in the homologous contralateral muscles. Improved knowledge will not only strengthen the hypothesis of cross-transfer effects, but also raise the awareness of the harmful consequences of such effects and increase opportunities for treatment and rehabilitation of many musculoskeletal disorders.

To test the hypothesis that there are bilateral morphological effects in the muscle tissue in response to marked one-sided overuse, we have used an experimental model of unilateral muscle overuse that affects the rabbit triceps surae muscle. The triceps surae consists of a pair of muscles, the soleus and gastrocnemius muscles, which have different molecular, metabolic and physiological characteristics. Both the soleus and gastrocnemius muscles of one leg were exposed to repeated electrical muscle stimulation and exercise (EMS/E) for three experiment periods, 1, 3 and 6 week. The muscles of both the exercised and contralateral non-exercised leg were analyzed with enzyme- and immunohistological methods, and the evaluations were focused on structural changes in the tissue and a possible inflammation.

## Materials and Methods

### Animals

A total of 24 female New Zealand white rabbits, aged from 6–9 months and having a weight of approximately 4 kg, were used in this experiment. The animals were divided into four groups consisting of six animals in each group. Three groups were exposed to the experimental exercise procedure on their right leg, and one group served as control. All animals were anaesthetized throughout the experiment by intramuscular injections of fentanylfluanison (0.2–0.3 ml/kg) and diazepam (0.2 ml/kg; 5 mg/ml). In order to maintain anesthesia, fentanylfluanison (0.1 ml/kg) was further injected every 30–45 minutes during the experimental procedure. To minimize pain after the experiment, buprenorphine (0.01–0.05 mg/kg), was given subcutaneously.

### Experimental Design

An experimental model using a kicking machine was used for the achievement of passive flexions and extensions of the right ankle joint. This model, which is a modified form of the model that was used by Backman et al [29], has previously been used for the induction of tendon changes (tendinopathy/tendinosis) in the Achilles tendon [30], [31]. The experimental model is described in detail in Backman et al [29]. The movements are produced by a pneumatic piston, in which the range of motion can be controlled. The range of movement was set to 9.5 cm, given a range of motion in the ankle of 55–65° of which 20–25° was dorsiflexion and 35–40° was plantarflexion. The right leg was attached to the piston and the pelvis/hip region was strapped down to restrict the motion in the left non-exercised leg. The left leg was unattached. During the plantar flexion of the right leg, an active contraction was induced by electrical muscle stimulation via surface electrodes (pediatric electrode 40 426A, Helwett Packard, Andover, MA, USA) placed 2 cm apart over the right triceps surae muscle. The stimulation was synchronized with the plantar flexion movement of the piston by a microswitch, which trigged the stimulator unit (Disa stimulator Type 14E 10; Disa Elektronik A/S, Herlev, Denmark). A single impulse with duration of 0.2 ms was delivered 85 ms after the initiation of the plantar flexion at an amplitude of 35–50 V. The stimulation intensity, which was tested out before the experiment, was submaximal and the intensity was individually corrected to obtain powerful muscle contractions. The electrical stimulus was controlled by an oscillometer and the frequency of the flexion and extension movements was set to 150 per minute (2.5 Hz) and the period for the repetitive movement was set to 2 hours. The experiment was repeated every second day and the total length of the experiment period was 1, 3 and 6 weeks. The frequency and duration of repetitive movements were chosen in order to make a very marked strain on the muscles. No reflex activity or co-contractions were visually observed in the contralateral non-exercised leg during the experiment period. In between the experiment periods, the rabbits were kept in ordinary cages, which allowed them freedom of movements. There was no visual signs that they favored the left non-experimental leg in between the experiment periods, nor that they showed amended movements or changed behaviors. All animals of each experimental group were sacrificed the day after the last experiment session by an intravenous overdose of Pentobarbital. This means that animals examined after an experimental period of 1 w had been subjected to the experimental sessions on four occasions. For further details, see [30], [31].

### Muscle Samples

Muscle specimens of an approximate size of 5×10 mm were taken from the distal portions of the soleus and gastrocnemius muscles of both legs. One specimen from each muscle was directly mounted in an OCT compound (Tissue Tek^®^, Miles Laboratories, Naperville, IL, USA) on a thin cardboard and rapidly frozen in liquid propane chilled with liquid nitrogen. An additional sample from the corresponding site in the muscles was immediately fixed in 4% formaldehyde in 0.1 M phosphate buffer, pH 7.0, for 24 hours at 4°C. After overnight washing at 4°C in Tyrode’s solution containing 10% sucrose, the chemically fixed samples were mounted and frozen as described above. Both the chemically fixed and the unfixed specimens were stored at −80°C until sectioning for microscopic analyses.

### Staining for Basic Histology

Muscle cross-sections, 7–8 µm thick, were cut in a cryostat microtome at –20°C and mounted on glass slides. The sections were stained with routine hemotoxylin-eosin (H&E) for demonstration of basic histology, including detection of degenerative and regenerative processes, inflammation and muscle fibers containing internal myonuclei [32].

### Immunohistochemistry

Five µm thick sections, serial to those used for H&E stain, were mounted on chrome-alum gelatin pre-coated glass slides and processed with previously well-characterized monoclonal antibodies (mAbs) against different types of white blood cells, nerve structures, glia cells and proteins that are related to muscle fiber degeneration, regeneration and necrosis. Macrophages were detected by using a monoclonal antibody (mAb) against glycoprotein CD68 (M0814, Dako, Denmark, diluted 1∶25) and neutrophils/T-cells were marked with a mAb against a cell surface antigen that is expressed by a subset of T-cells, thymocytes, and neutrophils (MCA805G, AbD Serotec, Oxford, UK, diluted 1∶100). Eosinophils were labeled with a mAb against eosinophil peroxidase (MAB1087, Chemicon, CA, USA, diluted 1∶100). Axons in nerves were stained with mAb T8660 against β-Tubulin (Sigma-Aldrich, USA, 1∶100) and Schwann cells were stained with mAb S2532 against S-100beta (β-subunit) (Sigma-Aldrich, USA, 1∶100). Motor-endplates in the neuromuscular junctions was marked with mAb MAB303 (Chemicon, CA, USA, diluted 1∶100), against acethylcholinesterase (AChE). Monoclonal Ab D33 (Dako, Denmark, diluted 1∶1000) against desmin and mAb 1891 (Chemicon, CA, USA, diluted 1∶200) against fibronectin were used for the detection of muscle fiber degeneration and fiber necrosis. Regenerative fibers were marked with mAb F1.652 (Developmental studies, Hybridoma Bank, USA, diluted 1∶50) against embryonic MyHC and mAb NCL-MHCn (Novacastra lab, Newcastle, UK, diluted 1∶200) against fetal MyHC. Visualization of the basement of the muscle fibers in the sections stained for developmental proteins were performed by double staining with mAb 5H2 against laminin α-2 chain (Novacastra lab, Newcastle, UK, diluted 1∶1000). All stainings were performed on sections of chemically fixed tissue or on post-fixed sections of unfixed tissue with the exception of mAb D33, mAb 1891, MAB303, F1.652, NCL-MHCn and 5H2 where sections of unfixed tissue were used. Post-fixation of the tissue was performed by incubating the sections for 10 min in 2% paraformaldehyde. The sections were then rinsed in phosphate-buffered saline (PBS).

Immunostaining was performed using standard techniques. The sections were first rinsed in PBS for 3×5 min and then incubated for 20 min in a 1% solution of detergent Triton X-100 (Kebo Lab, Stockholm, Sweden) in 0.01 M PBS, pH 7.2, containing 0.1% sodium azide as preservative. After this procedure the sections were rinsed 3×5 min in PBS and incubated in 5% normal rabbit serum in PBS supplemented with 0.1% bovine serum albumin (BSA) in PBS for 15 min in room temperature. All sections, with the exception of those stained with mAb T8660 against β-Tubulin (c.f. below), were thereafter incubated with the primary antibody diluted to appropriate concentrations in PBS with BSA in humid environment. Incubation proceeded for 60 min at 37°C or overnight at 4°C. After washes in PBS (3×5 min) and another incubation in normal rabbit serum, the sections were incubated with a secondary polyclonal Rabbit Anti-Mouse (TRITC) (nr 0276, Dako, Denmark), diluted 1∶40 in 0.1% BSA in PBS, for 30 min at 37°C. The sections were mounted in Vectashield hard set microscopy mounting medium (Dako, Denmark). In order to visualize the cell nuclei, Vectashield with DAPI was used (Dako, Denmark). For details of the laboratory procedures, see Forsgren et al [33] and Spang et al [34].

The staining procedure for mAb T8660 against β-Tubulin (Sigma, USA) differed from the scheme above. The sections were incubated with 5% normal goat serum and 5% normal horse serum and PBS containing 0.1% sodium-azide, 0.1% BSA and 0.2% Triton X-100 for 1 h at room temperature. They were then incubated with the primary antibody for 2 h at room temperature. After washes with PBS (2×30 min), another incubation in 5% normal goat serum and 5% normal horse serum followed. The sections were thereafter incubated with the secondary goat-anti-mouse antibody (Alexa Fluro 488®, diluted 1∶100) for 1 h at room temperature. After further washes, slides were mounted with coverslips using anti-fading Prolong Gold® with DAPI. For details of the laboratory procedures, see Tse et al [35].

For control of unspecific staining, sections were treated as described above, except that normal serum was used instead of primary antibodies. No specific staining was observed in these control sections. The specificities of the reactions obtained with the used antisera have previously been evaluated [34], [35].

### Morphological Analysis

Examination of the stained sections was carried out using a Zeiss Axioskop 2 plus microscope (Carl Zeiss, Oberkochen, Germany) equipped with an Olympus DP70 digital camera (Olympus Optical Co. GMBH, Hamburg, Germany). Analysis of histological changes was performed on sections stained with routine H&E. All calculations were made blinded to the origin of the samples. Evaluation of cells in the inflammatory infiltrates was made on sections stained with H&E and mAbs M0814, MCA805 and MAB1087. Estimation of fiber degeneration and fiber necrosis was based on fiber morphology and stainings for mAb D33 (desmin) and mAb 1891 (fibronectin). Muscle fibers infiltrated with inflammatory cells and with extensive intracellular reactivity for fibronectin and lack of staining for desmin were classified as necrotic fibers. Fibers with positive staining for developmental MyHCs, mAb A4.951 (embryonic MyHC) and mAb NCL-MHCn (fetal MyHC) and a basophilic staining in H&E were classified as regenerative fibers. Axon injury in the nerve fascicles was determined by negative staining for mAb T8660 and changes in Schwann cells appearances were analyzed on sections stained for mAb S100beta and DAPI.

### Statistical Analysis

All data are expressed as means and standard deviations (SD). A two way analysis of variance test (ANOVA) was used for analysis of differences in mean of each analyzed parameter in the three experimental groups and the controls. A multivariate analysis of variance test (MANOVA) was used to examine significant differences between the independent variables in each of the four groups. The test included analysis both within and between the exercised and non-exercised sides. The normality in residuals for each trait was examined and the distribution was normal or approximately normal except for one variable with flatter distribution. All the statistical analysis was performed by using the statistical software SAS/STAT vers, 9.2 (SAS Institute Inc, USA). A p-value <0.05 was considered to be significant.

### Ethics Statement

The study was approved by the ethical committee at Umeå University (protocol A34/07) that complied with national (SFS1988:534; 1988:539) and international (2010/63/EU) guidelines and standards in animal research. The approval was obtained before the start of the study. A licensed breeder had bred all the animals for the sole purpose of being used in animal experiments. All efforts were made to minimize animal suffering.

## Results

### General Findings

**Figure 1:**
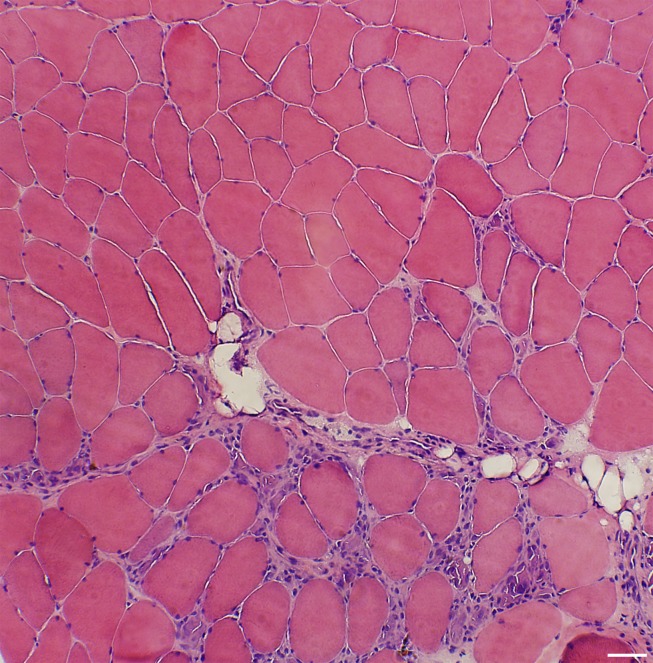
Localized histological changes in the muscle. Muscle sample stained with H&E from the exercised gastrocnemius muscle after 1 w of EMS/E. The figure shows a typical pattern of morphological changes and inflammation in local areas of the muscle tissue (bottom part). (Bar = 50 µm).

Our results show that the used experimental model caused severe muscle changes and inflammatory cell infiltration in focal regions of the exercised soleus muscles after 1 w of EMS/E. After 3 w and 6 w of EMS/E, structural tissue changes and myositis of various degrees were observed in restricted regions of the muscle samples in both the exercised and the contralateral non-exercised soleus and gastrocnemius muscles. The histological changes were mainly characterized by a marked fiber size variability, changes in fiber form, increased numbers of fibers with internal nuclei and fibers expressing developmental MyHCs, fiber splitting, fibrosis, and degeneration of axons in the nerve fascicles. A mild to severe accumulation of inflammatory cells was generally observed in the affected areas. Necrotic fibers and infiltration of fat were in some cases seen in the foci of the inflamed and fibrotic areas. The soleus muscle was generally more affected than the gastrocnemius muscle, although there was a large inter-individual variability in the severity of abnormalities within both muscles. The affected areas in all muscles were seen in the restricted regions of the samples (Fig. 1). No signs of inflammation or structural abnormalities were observed in the controls.

### Time Course and Magnitude of Histological Muscle Changes

In an attempt to quantify the magnitude of muscle changes and inflammatory cell infiltration in each muscle of the different experimental groups, the degrees of alterations in each sample were scored according to the criteria given in Table 1. The scored parameters were; degree of variability in fiber size (overall existence of small and large sized fibers), presence of fibrosis (abnormal presence of connective tissue), frequency of internal nuclei (percentage of fibers containing internal nuclei), frequency of fiber splitting (fiber splitting per mm^2^ muscle cross-sectional area), frequency of necrotic fibers (necrotic fibers per mm^2^ muscle cross-sectional area) and inflammatory response (degree of inflammatory cell infiltration). It should be noted that the scores of the histological changes to a high extent were related to the degree of changes that had occurred in focal areas of the muscle. The scores for each parameter in the different experimental groups are shown in Table 2.

**Table 1:** The principal basis for estimation of muscle changes and inflammation in the rabbit soleus and gastrocnemius muscle after unilateral EMS/E.

Scores	Variability inFiber size	Fibrosis	Fibers with internal nuclei (%)	Fiber splitting(fibers/mm^2^)	Necrotic fibers(fibers/mm^2^)	Inflammation
0	Normal	None	<2.5	<0.025	<0.025	None
1	Small	Mild	2.5–10	0.025–0.1	0.025–0.1	Mild
2	Moderate	Moderate	10–20	0.1–0.4	0.1–0.4	Moderate
3	Large	Marked	>20	>0.4	>0.4	Severe

The parameters used are variability in fiber size (overall existence of small and large sized fibers), fibrosis (abnormal presence of connective tissue), frequency of internal nuclei (percentage of fibers containing internal nuclei), frequency of fiber splitting per unit area (split fibers per mm^2^ muscle cross-sectional area), frequency of necrotic fibers per unit area (necrotic fibers per mm^2^ muscle cross-sectional area) and inflammation (degree of inflammatory cell infiltration in the muscle).

**Table 2:** Mean values of the scoring of variability in fiber size, fibrosis, percentage of fiber with increased internal nuclei, fiber splitting per unit area, presence of necrotic fibers per unit area and degree of inflammatory cell infiltration in the rabbit soleus (SOL) and gastrocnemius (GM) muscles in the exercised and non-exercised sides after 1 w, 3 w and 6 w of EMS/E.

	Variability in fiber size		Fibrosis		Fibers with internal nuclei		Fiber splitting		Necrotic fibers		Inflammation	
	Exercised	Non-exercised	Exercised	Non-exercised	Exercised	Non-exercised	Exercised	Non-exercised	Exercised	Non-exercised	Exercised	Non-exercised
**SOL**												
Control	–	0	–	0	–	0.5±0.5	–	0	–	0	–	0
1 week	1.4±0.5*	0.4±0.5	1.2±0.8*	0.6±0.5	1.4±1.1*	1.2±0.4	1.2±0.8*	0.6±0.9	1.8±0.8*	0	1.2±0.8*	0.4±0.5
3 week	1.2±0.4*	1.0±0.0*▪	1.3±0.5*	0.8±0.4*	1.3±0.5*	1.3±0.5*	0.8±1.0*	0.8±0.4*	0.8±1.0*▪	0.3±0.5	1.3±0.8*	0.8±0.4*
6 week	2.2±0.8*^▴△^	2.8±0.4*^▴△^	2.0±0.9*▴	2.0±0.6*^▴△^	2.5±0.5*^▴△^	2.8±0.4*^▴△^	2.2±0.8*^▴△^	2.2±0.8*^▴△^	1.7±1.2*	1.8±0.8*^▴△^	2.0±0.9*▴	1.8±0.8*^▴△^
**GM**												
Control	–	0.2±0.8	–	0	–	0.7±0.5	–	0	–	0	–	0
1 week	0.5±0.8	0.2±0.4	0.5±0.8	0.7±0.5	1.2±0.4	1.0±1.0	0.2±0.4	0.3±0.8	0.3±0.8	0.3±0.8	0.5±0.8	0.3±0.5
3 week	1.8±1.0*▪	1.5±0.8*▪	1.7±1.0*▪	1.2±1.0*	1.7±1.0*	1.5±1.2	1.5±1.4*▪	0.8±1.3	1.0±1.5	0.8±1.3	1.5±1.2*▪	0.6±1.2
6 week	1.3±0.8*	1.3±0.8*▴	1.0±.0*	1.2±1.0*	1.7±1.0	1.5±0.9*	0.5±0.8△	1.2±1.1*	0.3±0.5	0.7±0.8	0.3±0.5▴	0.8±0.7*

The values are presented as mean ± SD. Significant differences (p<0.05) are marked; * to controls,

▪ 3 w *v.s.* 1 w group,

▴ 6 w *v.s.* 1 w group and,

△ 6 w *v.s.* 3 w group.

### Soleus Muscle

#### Controls

The muscles of the control animals had a muscle morphology characterized by densely packed polygonal fibers of about similar sizes. Only occasional fiber splitting, necrotic fibers, fibers containing developmental MyHCs and inflammatory cells were found. The mean number of fibers with internal nuclei was below 2.5% of the total fiber population.

#### Exercised side

**Figure 2:**
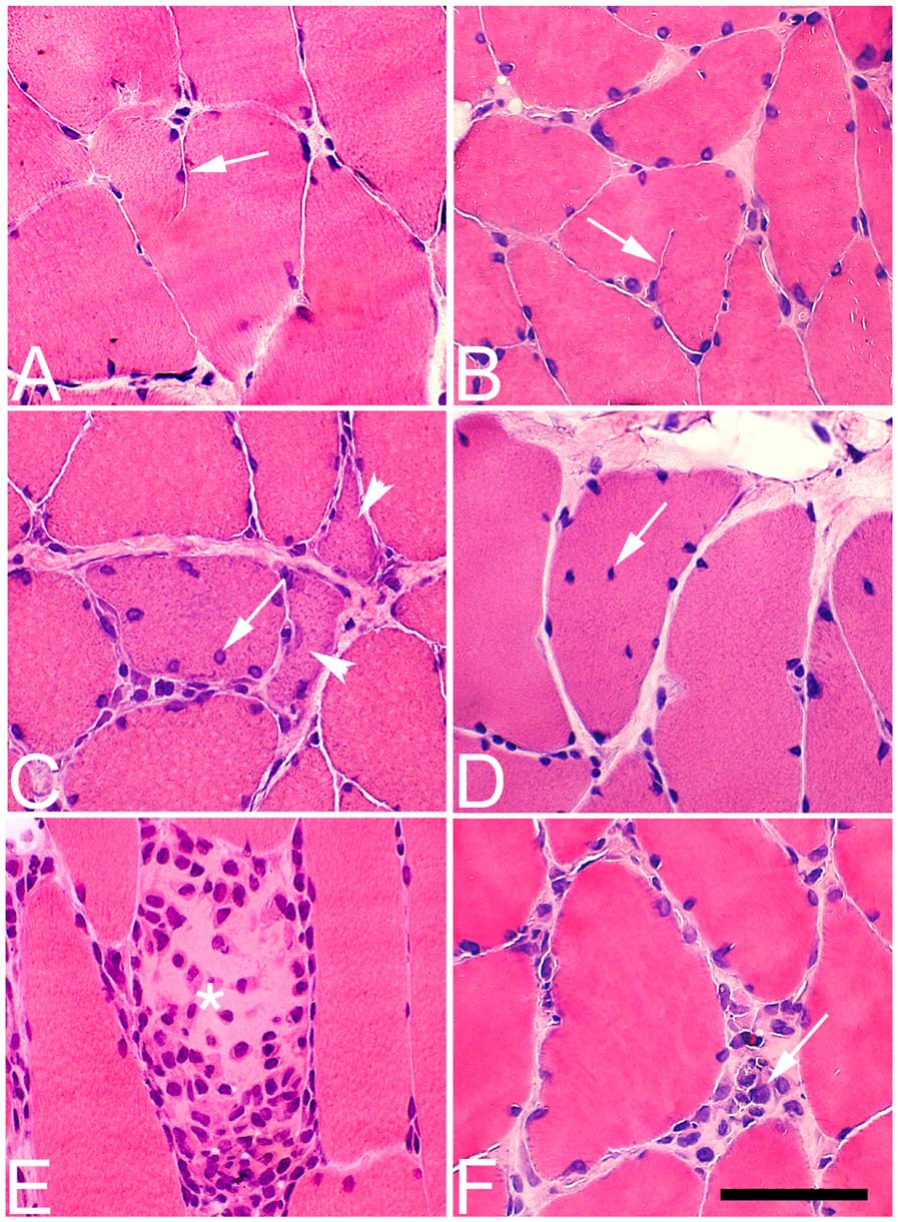
Histological changes in the soleus muscle after 1 w. Muscle samples from the exercised side (left column, A, C, E) and contrateral non-exercised side (right column, B, D, F) of the soleus muscle after 1 w of EMS/E. The sections are stained with H&E. The left column (exercised side) shows fiber hypertrophy and fiber splitting (arrow) (A), small angular fibers (arrowheads) (C) and existence of internal nuclei (arrow) (C) and inflammatory cell infiltration in the area of a necrotic fiber (asterisks) (E). The right column (non-exercised side) shows occurrence of fiber splitting (arrow) (B), internal nuclei (arrow), fiber hypertrophy (D) and an accumulation of inflammatory cells in the extracellular matrix (arrow) (F). (Bar = 50 µm).

**Figure 3:**
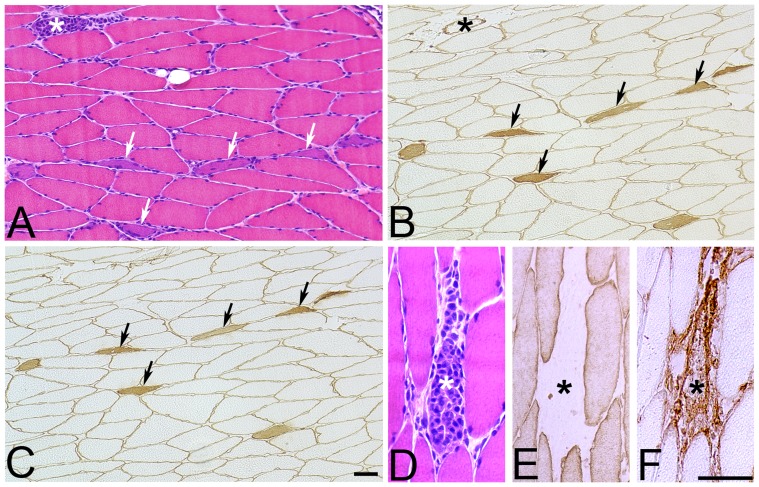
Muscle fiber regeneration and degeneration. Serial sections from a exercised soleus muscle after 1 w of EMS/E. The sections are stained with H&E (A, D) and for embryonic MyHC (B), fetal MyHC (C), desmin (E) and fibronectin (F). Sections B and C are double stained for Laminin α-2 chain for visualization of the basement membrane of muscle fibers. Figures (A–C) show regenerating fibers (arrows) and a necrotic fiber (asterisks) (A, B). This necrotic fiber is shown in figures (D–F). Note the infiltration of inflammatory cells in the necrotic fiber (D), the lack of reactivity for desmin (E) and the extensive reactivity for fibronectin (F) in this fiber (Bar = 25 µm).

**Figure 4:**
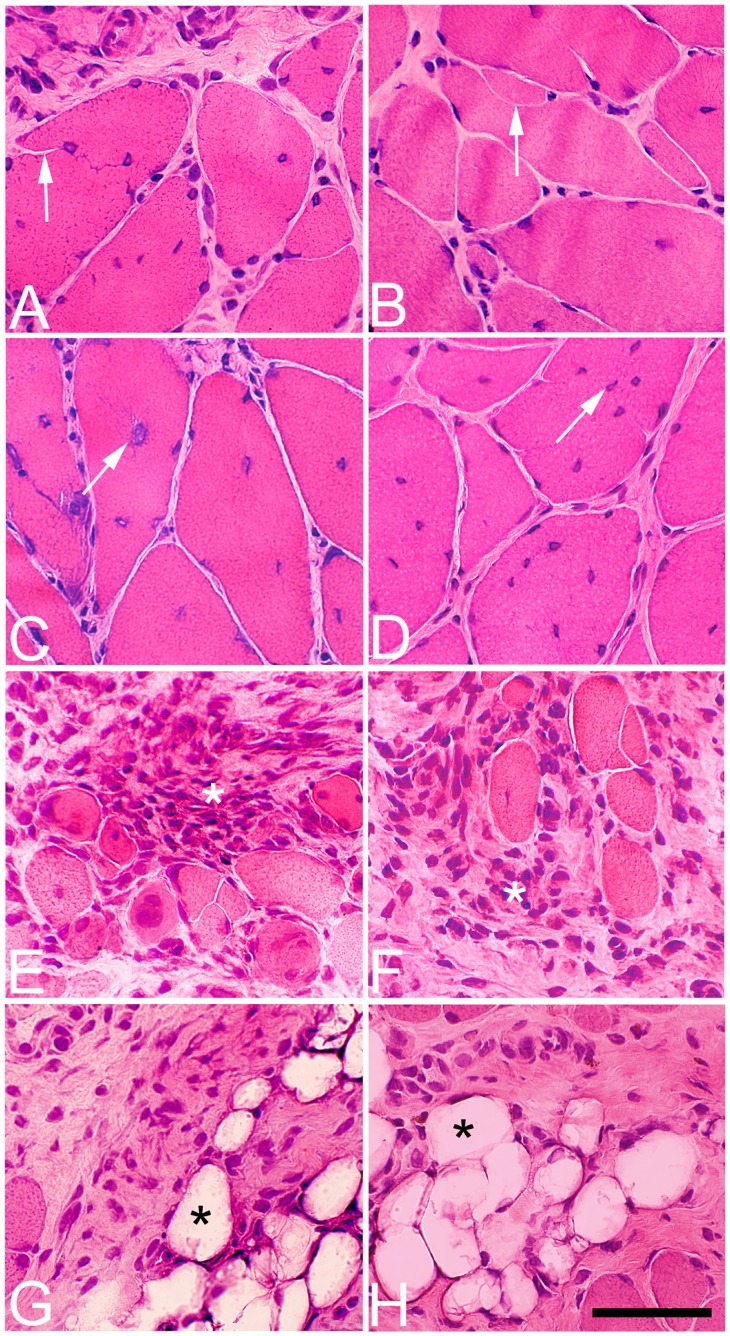
Histological changes in the soleus muscle after 6 w. Muscle samples from the exercised (left column, A, C, E, G) and non-exercised (right column, B, D, F, H) soleus muscle after 6 w of EMS/E. The sections are stained with H&E. Note the high variability in fiber size (A–H), the presence of fiber splitting (arrows) (A, B), multiple numbers of internal nuclei (arrows) (C, D), fibrosis and infiltration of inflammatory cells (asterisks) (E, F) and fat infiltration (asterisks) (G–H) in both the exercised and non-exercised muscles. (Bar = 50 µm).

After 1w of EMS/E, several structural changes were found in the muscle tissue (Table 2). An increased variability in fiber size, fibrosis, fiber splitting, necrotic fibers, and fibers containing developmental MyHCs could occasionally be seen in focal areas of the muscle (p<0.05) (Fig. 2 and 3). A mild infiltration of inflammatory cells was found in four of the cases and fibers with internal nuclei were observed in some inflamed areas (p<0.05). In the 3 w group, the structural changes were to some extent of the same magnitude as seen after 1 w. After 6 w of EMS/E, the degree of histological changes had increased and all parameters had higher scoring than in controls and to some extent also as compared to 1 w and 3 w groups (P<0.05) (Table 2). At this stage, inflammatory cell infiltration was found in all cases, with a mild to moderate inflammatory response being observed in focal areas of five cases and a severe focal inflammatory reaction in one. In the latter case, the inflamed areas contained large numbers of necrotic fibers, fiber splitting and fibers with internal nuclei (Fig. 4). Fibrosis was common in the 6 w group and some samples also exhibited fatty infiltration.

A multivariate analysis of variance test for all parameters showed that the morphological changes were higher for all three experimental groups compared to the controls (p<0.001). The changes were higher in the 6 w group compared both the 3 w and 1 groups (p<0.0004).

#### Non-exercised side

After 1w of EMS/E, a few histological changes were observed, although the scores for each parameter did not reach statistical significance. A mild infiltration of inflammatory cells and fiber splitting was locally seen in two of the cases (Fig. 2). There was a tendency of an increased number of fibers with internal nuclei in all cases and some fibers contained developmental MyHCs. After 3 w, the mean scores for fiber size variability, fibrosis, fibers with internal nuclei, fiber splitting, and inflammatory cell infiltration were increased compared to controls (p<0.001) (Table 2). The number of fibers expressing developmental MyHCs was generally higher than at 1w. Necrotic fibers were found in two cases. At 6 w, the scored mean values for all parameters and number of fibers containing developmental MyHCs were significantly increased as compared to controls (p<0.001) and fatty infiltration was observed in restricted areas of some samples. The severity of inflammatory cell infiltration was also more marked compared to the 1 w and 3 w groups (P<0.05) (Table 2). There was a severe inflammatory cell infiltration in two of the cases, and within the inflamed areas, there was also a large number of necrotic fibers, fiber splitting, fibers with internal nuclei and fatty infiltration (Fig. 4).

The multivariate analysis test showed that the scores of the parameters were significantly higher in each experimental group compared to the controls (p<0.05). The differences was significantly higher at 6 w than at 3 w, which in turn was higher than at 1 w (p<0.001).

#### Comparison between exercised and non-exercised side

The multivariate test revealed higher changes in the scores of the used parameters in the exercised than non-exercised muscles after 1 w of EMS/E (p<0.001). After 3 w and 6 w of EMS/E, the scores increased in the same range for both sides.

### Gastrocnemius Muscle

#### Controls

The muscle was composed of densely packed polygonal formed muscle fibers of about similar sizes. Splitting fibers, necrotic fibers, fibers containing developmental MyHCs and inflammatory cells were absent or very rare. The mean number of fibers with internal nuclei was below 2.5% of the fiber population.

#### Exercised side

**Figure 5:**
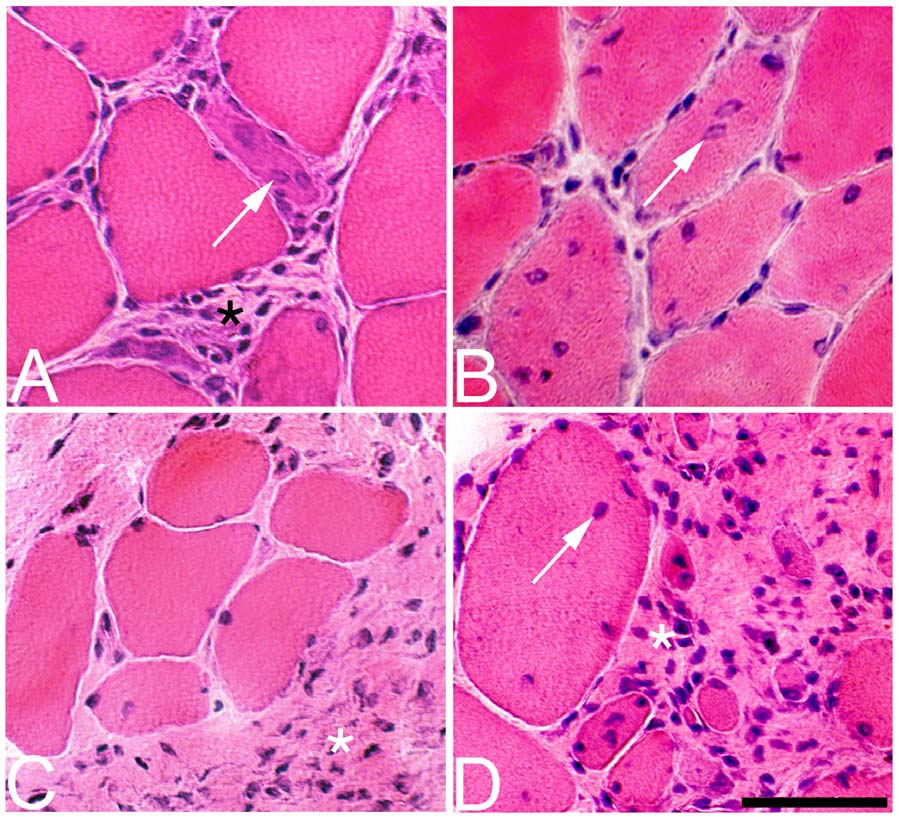
Histological changes in the gastrocnemius muscle after 1 **w and 6** **w.** Muscle samples from the exercised (left column, A, C) and non-exercised contralateral (right column, B, D) sides of gastrocnemius muscles after 1 w (A, B) and 6 w (C, D) of unilateral EMS/E. The sections are stained with H&E. Note the presence of atypical muscle fibers indicating muscle fiber regeneration in the exercised muscle (A) and fibers with internal nuclei (arrow) in the non-exercised muscle (B) after 1 w of EMS/E. Note also the large variation in the fiber size (A–D), the presence of small rounded fibers (C, D) and the marked fibrosis and inflammatory cell infiltration with extended duration of EMS/E (asterisks) (A, C, D) in both the exercised and non-exercised sides. Figure B and D shows presence of internal nuclei (arrows). (Bar = 50 µm).

After 1 w of EMS/E, no parameters differed significantly from those of the controls (see Table 2). However, two cases exhibited a marked variability in fiber size, a mild fibrosis and an increased number of fibers with internal nuclei in local areas of the muscle sample (Fig. 5). One case contained focally a large infiltration of inflammatory cells and a high number of fiber splitting and necrotic fibers. At 3 w, the scores for all parameters except fiber necrosis were significantly higher compared to controls (p<0.01) (Table 2). Two cases had severe changes for all parameters examined and the number of muscle fibers containing developmental MyHCs were generally increased. After 6 w of EMS/E, the morphological changes were in the same magnitude as after 3 w of EMS/E, with the exception that the degrees of fiber split and inflammation were lower (p<0.02) and some cases had now infiltration of fat in the affected areas.

The multivariate analysis showed that the changes of the parameter scores were higher in the experimental groups at both 3 w and 6 w compared to the controls (p<0.002). At 6w the changes were reduced compared to 3 w (p<0.001).

#### Non-exercised side

After 1w of EMS/E, there was no significant difference in any parameters compared to the controls (Table 2). However, in one case the scores for all parameters were relatively high. The specimen of this case exhibited focal areas with inflammatory cell infiltration and within the affected areas there was a high number of both fiber splitting and necrotic fibers and fibers with frequent internal nuclei (Fig. 5). After 3 w of EMS/E, there was a higher variability in fiber size, an increased number of fibers expressing developmental MyHCs, and a larger amount of connective tissue than in controls (p<0.01). Two cases had marked morphological changes in the muscle tissue. At 6 w, the severity of changes was significantly different compared to controls for all parameters except for fiber necrosis. Some samples at this stage had infiltration of fat in the muscle tissue.

The multivariate analysis test for the used parameters showed that the changes were higher at 3w compared to the controls (p = 0.02). At 6 w of EMS/E, the values for each parameter showed a trend against significant changes compared to controls (p = 0.06).

#### Comparison between exercised and non-exercised sides

The multivariate test revealed no differences between the exercised and non-exercised sides after 1 w of EMS/E. At 3 w, the values for the used parameters were higher in the exercised side than in the non-exercised side (p = 0.002). After 6 w of EMS/E, the values were lower in the exercised side compared to the non-exercised side (p = 0.02).

#### Changes in nerve tissue

**Figure 6:**
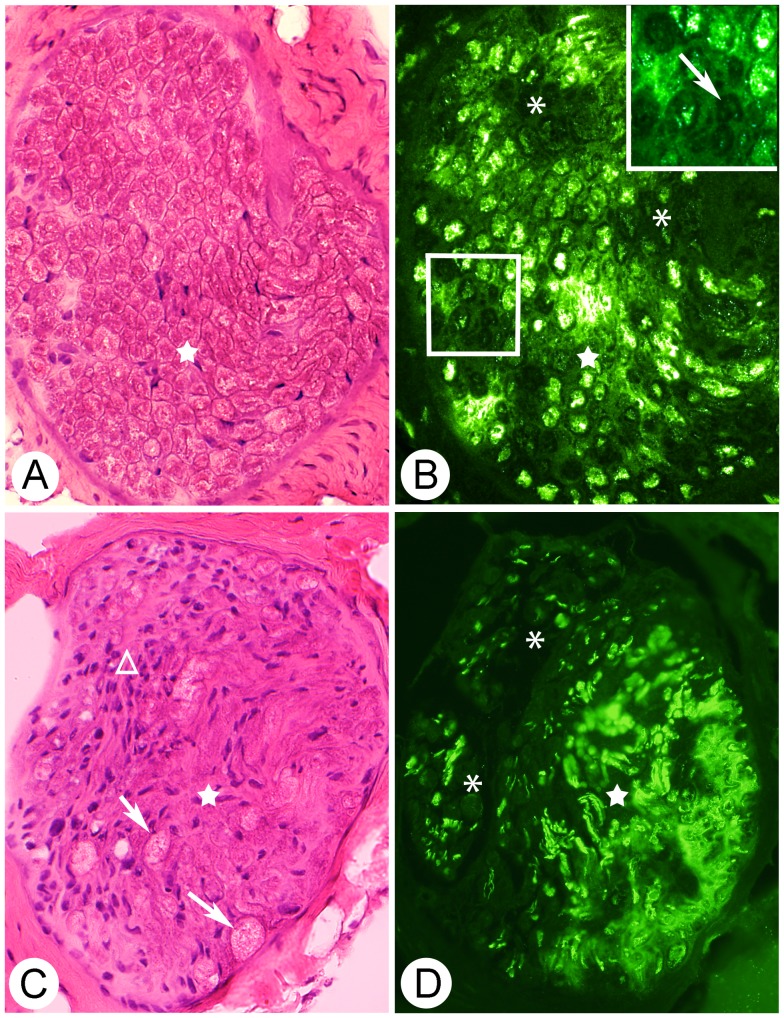
Serial sections of nerve fascicles. Cross-sections of nerves fascicles from the non-exercised side of the soleus muscle after 6w of EMS/E. The sections are stained with H&E (A, C) and for β-Tubulin (mAb T8660) (B, D). Framed region in (B) is inserted in larger magnification (top right). Stars show the corresponding area in (A, B) and (C, D). Note the weak or non-existing β-Tubulin immunoreaction for some axons in (B) and (D) (asterisks, marked with arrow in framed region). In (C), ballooned foamy cell structures are marked (arrows). Note also the fibrotic appearance and the presence of a large number of cell nuclei in the nerve fascicle in (C), especially in the area marked with a triangle. (Original magnification x200).

After 1 w of EMS/E, the histological appearance within nerve fascicles appeared normal. However, after 3 w, and especially after 6 w of EMS/E, histological changes could be observed in some nerve fascicles located in the affected areas in the soleus and gastrocnemius muscles of both the exercised and non-exercised sides (Fig. 6). The changes within the nerve fascicles observed with the H&E stain corresponded to increased numbers of cell nuclei, increased amount of connective tissue and an occurrence of cellular structures with a ballooned, “swollen” and foamy cytoplasm (Fig. 6).

**Figure 7:**
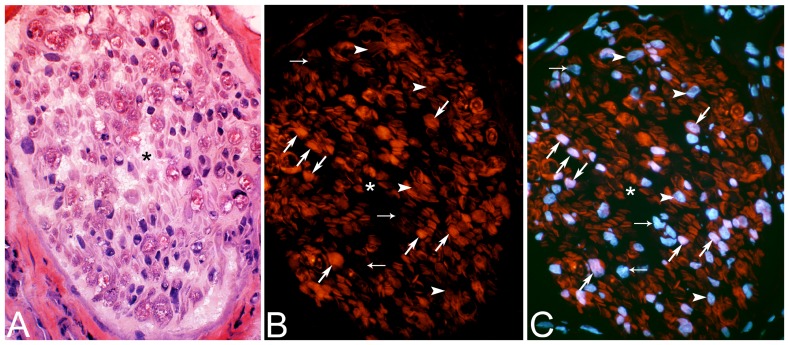
Serial sections of nerve fascicles. Cross-sections of a nerve fascicle from soleus muscle (non-exercised side) after 6w of unilateral EMS/E. The sections are stained with H&E (A), mAb S-100beta (B) and mAb S-100beta and DAPI (C). Note the marked presence of connective tissue and a high number of cell nuclei within the nerve fascicle (A). In (B), mAb S-100beta stains Schwann cells. Figure (C) shows two patterns of stained nuclei, one bluish and one pink, where the bluish nuclei are stained only for DAPI. Some of the nuclei located in cell cytoplasm are devoid of S-100beta reaction (small arrows) whilst others nuclei in the cytoplasm exhibits a S-100beta reaction around the nuclei (arrowheads). The cells with pink nuclei showed immunoreaction in the cytoplasm for mAb S-100beta (large arrows). (Original magnification×200).

The immunohistochemical staining with the β-Tubulin antibody (mAb T8660) showed that a subpopulation of axons was unstained in the nerve fascicles with an abnormal morphology in the soleus and gastrocnemius muscles of both legs after 3 and 6w of EMS/E (Fig. 6). In comparison, all axons within nerve fascicles in the controls and the 1w groups showed staining reactions for this mAb. Examination of sections stained with DAPI verified that there was an increased number of nuclei within these nerve fascicles. In the sections stained with the mAb S-100beta where DAPI was used in the mounting medium, the nuclei population showed two patterns of staining. One population showed a characteristic bluish DAPI reaction that usually is observed for Schwann cell nuclei, and the other exhibited a pink staining reaction (Fig. 7). All pink stained nuclei showed staining reaction for both DAPI and the mAb S-100beta. The cytoplasm of some of the cells with bluish stained nuclei showed S-100beta reaction, while others lacked S-100beta reaction. The latter cells are presumably fibroblasts or smooth muscle cells, whereas the cells with cytoplasmic reactions with mAb S-100beta are considered as Schwann cells. The staining for white blood cell markers (c.f. below) showed that only occasional white blood cells were detected within the nerve fascicles and these cells exhibited reactions for the neutrophil/T-cells antibody.

#### AChE immunoreaction

**Figure 8:**
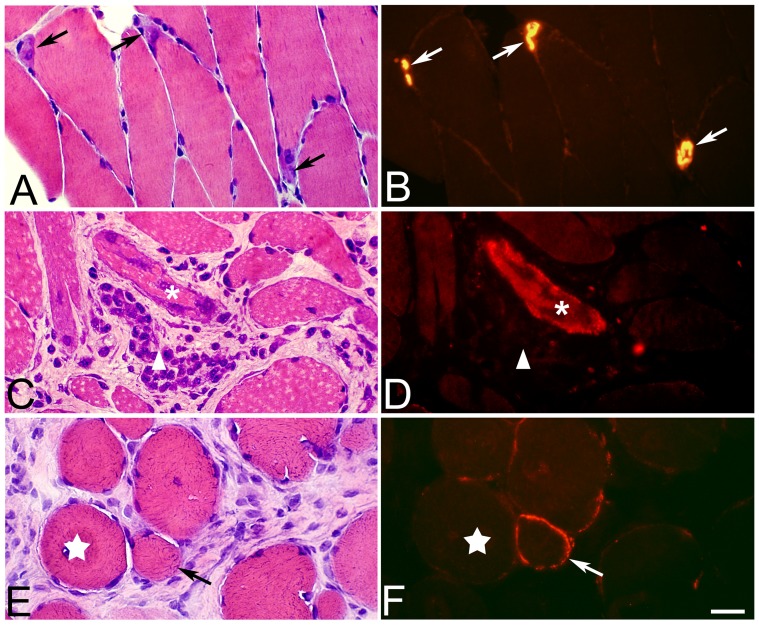
AChE reactivity pattern. Serial sections from a control (A, B) and a non-exercised (C, D) and exercised (E, F) soleus muscle after 6w of EMS/E. The sections are stained with H&E (A, C, E) and for AChE (B, D, F). A typical staining pattern for AChE in motor-endplates is shown in the control muscle (B) (arrows at corresponding locations in A and B). Figures D and F show an atypical AChE staining pattern of muscle fibers. Note the high AChE activity in the regenerating fiber in figure (D) (asterisks) and the AChE reaction on the surface of a fiber with normal morphology (arrow) in (F). A necrotic fiber is marked with an arrowhead in figures (C, D). Star marks similar fiber in the cross-sections. (Bar = 25 µm).

The immunohistochemical staining reaction for AChE showed a normal pattern for motor endplates in the neuromuscular junctions in both controls and in the 1w experimental groups. After 3w and 6w of EMS/E, a number of fibers in the affected regions of both legs showed an abnormal AChE staining pattern where the AChE reactivity encircled a large part of the muscle fibers (Fig. 8). This type of reaction was observed in both fibers that had a normal morphological appearance as well as fibers with an abnormal appearance. No AChE staining was observed in necrotic fibers.

#### Types of white blood cells

**Figure 9:**
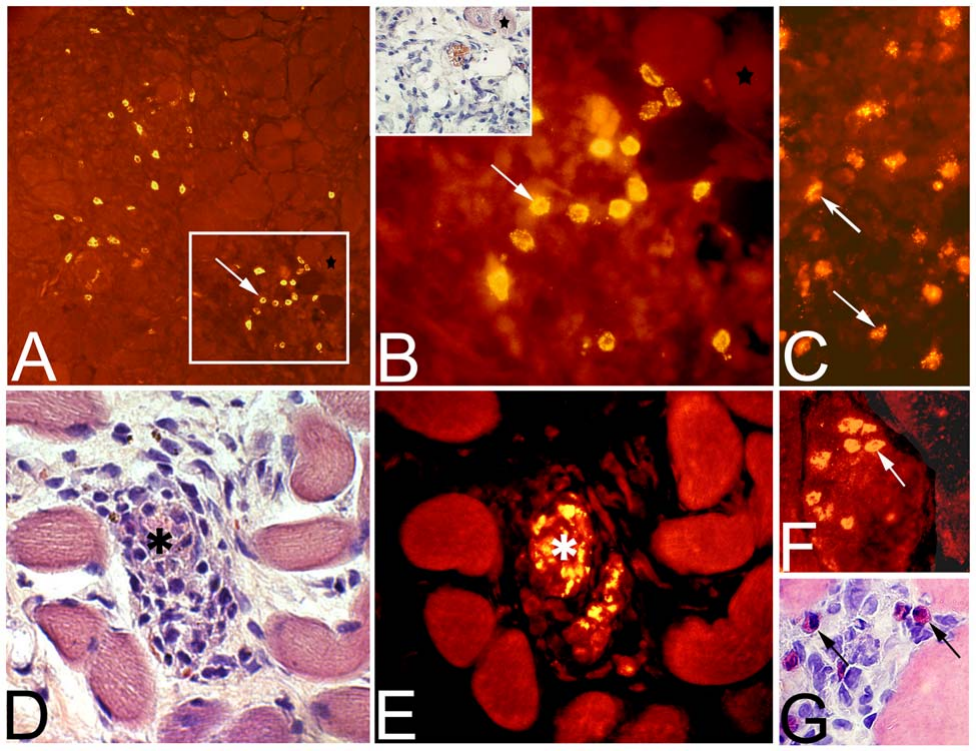
Staining for white blood cells. Muscle cross-sections from the non-exercised soleus muscle after 6w of EMS/E (A, B, D–G) and from the exercised side of the gastrocnemius muscle (C). The sections A–C are stained for demonstration of neutrophils/T-lymphocytes (mAb MCA805G). The framed region in (A) is in higher magnification in (B) and the parallel section to the framed region stained for H&E is in the inset (B). The figures show a large number of immuno-reactive cells (arrows) in the connective tissue (A–C). Stars show corresponding muscle fiber in (A) and (B). Figure D show infiltration of inflammatory cells in a necrotic fiber stained with H&E and figure E show the corresponding necrotic fiber (asterix) stained with mAb M0814 against CD68 (macrophages). Figure F show eosinophils (mAb MAB1087) infiltrating a muscle fiber (arrow) In (G), eosinophils (arrows) are stained with H&E in the extracellular matrix of another region of the muscle sample. (Original magnification; A ×100, B–E ×200, F and G x315).

The immunohistochemical analysis showed that cells in the inflamed areas of the muscle cross-sections were stained by the antibodies directed against macrophages (CD68, mAb M0814), neutrophil/T-cells (mAb MCA 805G), and eosinophils (MAB 1085) (Fig. 9). CD68 stained cells were generally observed within and around necrotic fibers (Fig. 8), while the cells demarcated by the neutrophil/T-cell antibody were observed around damaged muscle fibers and in areas with fibrosis (Fig. 9). Cells stained with the mAb against eosinophils were generally widely spread over the affected areas in the muscle and some stained cells were found within atypical or affected fibers (Fig. 9).

## Discussion

In this study we have used an experimental model to cause unilateral muscle overload in the triceps surae muscle in response to passive flexion/extension movements of the ankle joint combined with an active contraction in the flexion phase via electrical stimulation. The most interesting result of this experiment was that the repetitive unilateral EMS/E caused histological changes and inflammation in the muscle tissue not only in the exercised muscles, but also in the contralateral muscles in the non-exercised leg.

### Time Pattern of Muscle Changes

After 1w of EMS/E, the exercised soleus muscle showed several structural alterations in the muscle tissue, whereas only a few changes were found in the exercised gastrocnemius muscle. At this time, there were only limited modifications of the tissue in the homologous contralateral non-exercised muscles. After 3w of EMS/E, significant histological changes of the muscle tissue were found bilaterally in both the soleus and gastrocnemius muscles. The magnitude of these abnormalities increased significantly in both sides from 3w to 6w of EMS/E in the soleus muscles, while the changes in the gastrocnemius muscles at this time were more or less in the same magnitude as in the 3w group. These observations imply that the magnitude of alterations due to EMS/E follows a specific time sequence, that there is a delay in the cross-transfer effects to the contralateral side and that the soleus muscle is more susceptible to influence than the gastrocnemius muscle for this type of experimental exercise. The cause of the more severe abnormalities in the soleus than in the gastrocnemius muscle is unclear, but one explanation might be the characteristic differences between the muscles in fiber phenotype composition, i.e. the soleus muscle is mainly composed of slow twitch MyHCI fibers whereas fast twitch MyHCII fibers predominate in the gastrocnemius muscle [36]. A more detailed study of the effect of EMS/E on fiber phenotype composition and fiber size is in progress in our laboratory.

### Changes in Nerve Fascicles

Morphological nerve changes were observed in both exercised and non-exercised soleus and gastrocnemius muscles after 3w and particularly after 6w of EMS/E. A subpopulation of axons in some nerve fascicles in the affected muscle areas was unstained for β-Tubulin, indicating axonal degeneration (Fig. 6). Furthermore, within these nerve fascicles there was an increased content of connective tissue and a high number of cell nuclei. Interestingly, staining of the affected nerve fascicles for mAb S-100beta in combination with DAPI showed that a subpopulation of the Schwann cell nuclei revealed reactions for both these markers (Fig. 7). In the normal nerve fascicles, DAPI stains all nuclei, whereas the S-100beta protein generally is restricted to the cytoplasm and the membranes of the Schwann cells [37]. It is thus possible that the nuclei that were stained by both markers represent Schwanns cells in a proliferative phase. This assumption is based on reports that Schwann cells are activated in response to nerve damage and that the S-100beta protein plays crucial roles in axonal repair and regeneration and CNS development [38], [39], [40]. The high number of Schwann cell nuclei in the affected nerve fascicles and the S-100beta reaction in some of these nuclei supports proliferation of Schwann cells within the nerve fascicles (Fig. 7). These findings imply that there is a bilateral process of regeneration and repair of injured nervous tissue in the muscles.

### AChE Reaction Pattern

Normally AChE activity is restricted to a small area on the muscle fibers that represents motor-endplates in the neuromuscular junctions. In this study, we observed that after 3w, and especially after 6w of EMS/S, some muscle fibers in the affected areas of both the exercised and non-exercised sides had an abnormal staining pattern for AChE where the fibers were more or less encircled by AChE reactivity (Fig. 8). It is previously shown that myofibers of denervated and regenerating fibers can produce AChE and that regenerating motor nerves hereby play an indirect role by inducing the myofibers to produce synaptic AChE [41]. Our finding supports affections of the motor-nerve innervations within the focal areas and imply that a subpopulation of fibers in both the exercised and non-exercised sides are in a regenerative stage.

### Muscle Tissue Changes

The bilateral histological muscle changes in the muscle tissue were characterized by focal areas with increased variability in fiber size and fibrosis and within these areas there were, apart from an inflammatory infiltration, a high number of fibers with internal nuclei, fiber splitting and necrotic fibers (Figs. 2, 4, 5).

Skeletal muscle is a stable tissue with little turnover of nuclei. However, after injury, e.g. due to extensive physical exercise, skeletal muscle has the ability to go through a rapid and extensive regeneration. The initial event of the regenerative process is fiber injury and necrosis, followed by activation of mononucleated cells i.e. an inflammation. This phase is followed by activation of myogenic cells to proliferate, differentiate and fuse, leading to new fiber formation and reconstruction of damaged tissue [42]. Our findings of bilateral histological muscle changes in focal areas suggest that the performed experiment causes a bilateral process of fiber degeneration and fiber regeneration in the muscles.

In view of the signs of axonal degeneration, it is possible that the marked presence of small-sized fibers relates to denervation atrophy or to newly formed fibers originating from activated satellite cells [43]. The expression of developmental MyHCs in some small sized fibers and presence of atypical formed fibers with a basophilic staining (reflecting high protein synthesis) and central nucleus further supports a regenerative process with newly formed fibers in the muscles (Fig. 3). The concomitant presence of abnormally large fibers (Fig. 5) suggests muscle fiber regeneration due to a frequent activation of certain motor-units or an up-regulation of other regenerative processes via chemical signals by growth factors [1]. The mechanism behind the increased numbers of fiber splitting and fibers with internal myonuclei after EMS/E is unclear, but both of these features are common findings in connection with muscle hypertrophy due to muscle overload [44] and in neuromuscular disorders [45]. The mechanisms responsible for fiber splitting can be related to fusion of activated and multiplying satellite cells to existing damaged fibers or to disturbed regeneration after segmental muscle fiber injury [46]. An incomplete fusion of fibers regenerating within the same basal lamina is considered to be a characteristic feature of muscle regeneration. The internalization of myonuclei is suggested to be an outcome of this process [46]. Once fusion of myogenic cells is completed, newly formed fibers increase in size, and internal myonuclei move to the periphery of muscle fiber [42]. A higher number of nuclei in fibers could thus be causally related to an upregulation of protein synthesis capacity of the fibers [44].

### Inflammation

In the exercised soleus muscles, we found a focal and mild accumulation of inflammatory cells in the muscle tissue already after 1w of EMS/E (Fig. 2). After 3w of EMS/E, a distinct myositis was present in local areas of both the exercised and non-exercised soleus and gastrocnemius muscles. At 6w, the inflammatory process had further increased in the soleus muscles of both legs, but not in the gastrocnemius muscles. It is evident that the inflammatory cell infiltration increased with the duration of EMS/E in the soleus muscle, whereas it peaked after 3w in the gastrocnemius muscle.

The first event that occurs after muscle damage is an invasion at the injury site by inflammatory cells. The inflammatory process is a part of the complex biological response to harmful stimuli. There is also some evidence that the nervous system can be involved in the inflammatory processes. Denervation of joints leads to regression of established rheumatoid arthritis (RA) and protection of development of RA [47], [48] and psoriasis improves following skin denervation [49].

The inflammatory process is normally time-dependent; neutrophils rapidly invade the muscle tissue, later followed by macrophages, cells known to be phagocytic [50]. Of the inflammatory cells detected in this study, macrophages and neutrophil/T-cells were commonly accumulated within the focal areas with severe histopathological muscle changes, whereas eosinophils usually were more widely spread in the affected areas (Fig. 9). Some necrotic or damaged muscle fibers were invaded by phagocytic macrophages. Macrophages have been shown to promote muscle injury through the release of free radicals, but they also seem to participate in muscle repair and regeneration [51]. Previous studies have shown that phagocytic neutrophils invading into muscle tissue have the ability to release proteases for the removal of debris related to the injury and to produce high concentrations of cytolic and cytotoxic molecules that can further damage the muscle tissue [52], [53]. The degrading factors from the inflammatory cells may cause additional cell injury, which may contribute to the accumulation of histopathological muscle changes in the inflamed areas.

### Fibrosis and Fat Infiltration

It is well known that the type of inflammatory response after muscle injury crucially can influence the outcome of muscle repair, or alternatively, fibrosis [51], [54], [55], [56]. Fibrosis is essentially an excessive accumulation of extracellular matrix components, particularly collagen, which is the end result of a cascade of events proceeding from tissue damage via inflammation [57]. It is known that when regeneration of the muscle tissue does not occur, the fibrotic tissue can also be infiltrated with adipocytes, causing fatty infiltration [58]. The findings of increased fibrosis and fatty infiltration with increased experimental length indicate that the repetitive EMS/E model not only causes injury and myositis but also an imbalance in the repair process with increased connective tissue synthesis and fat infiltration in both the exercised and non-exercised legs (Fig. 4).

### Cross-transfer Effects

The qualitative similarities in tissue changes and inflammation in the exercised and non-exercised muscles after 3 and 6w of EMS/E show that there is a symmetric process. This observation is in line with previous findings that indicate that there is a signaling system across the midline of the body [1]. Following peripheral nerve lesions there are well-documented events that affect the contralateral nonlesioned structures. Unilateral section of a motor nerve can for example cause robust signs of sprouting at the neuromuscular junction of the collateral homologous muscle [1]. Other cross-transfer effects of the nervous system related to the locomotors system is the remarkably symmetrical distribution of inflammation in some chronic inflammatory diseases [10]. The symmetry of inflammation in RA is probably mediated by stimuli-specific responses of the sensory nerves, i.e. a unilateral pro-inflammatory stimulus causes a contralateral reaction [9], [10]. Previous studies also indicate that there is a mirror image of the nerve signal system for pain [11], [12]. Interestingly, a recent study on the effects of treatment with mini-invasive scraping technique for patients with bilateral Achilles tendinopathy showed that unilateral treatment mostly lead to curing of the chronic pain also on the non-treated side [15]. A sensory cross-over neuronal mechanism was suggested to be responsible for the contralateral effects on the non-operated side. Another important finding supporting cross-talk of the nervous system is the previous reports of increased strength in the homologous contralateral muscle after unilateral exercise [2], [3], [4]. Nevertheless, the mechanism behind the severe bilateral muscle damage and inflammation after unilateral EMS/E, as seen in the present study, and the reports of increased muscle strength may not be the same. Hypothetically the increased strength in the contralateral limb is akin to a process of motor learning, i.e motor areas in the brain that are responsible for motor control have adapted to unilateral voluntary training and the opposite hemisphere in the brain may have access to these modifications [4], [8]. Alternatively, unilateral training might enhance the organization of the spinal and cortical motor pathways to the contralateral limb which results in an increased drive to the untrained limb [8]. The collateral muscle changes and inflammation after unilateral EMS/E observed in this study may be caused by another neuronal mechanism. Since there is some evidence for a commissural system in the spinal cord that mediates transmedian signaling with a fairly precise bilateral representation [1], nerve signals from the trained side may pass over to the contralateral muscles through commisural inter-neurons. If this is the case, unilateral injury caused by EMS/E may cause a cross-transfer up-regulation of neuropeptides that can be involved in the inflammatory response in the contralateral muscles [59]. Preliminary studies in our laboratory indicate that this is the case in our experimental model. However, further studies are needed to elucidate the precise mechanism to the contralateral effects. Nevertheless, since some cross-transfer effects may be harmful and others useful in rehabilitation/treatment, the occurrence of contralateral processes may be important to consider in clinical situations in a wide range of musculoskeletal and neuromuscular disorders.

The contralateral responses observed in the muscles, including the nerve tissue, could hypothetically be mediated through systemic or circulatory effects. However, since the histological changes and the myositis occurred only focally and not generally in the muscle tissue, it is unlikely that the main part of the changes can be related to a systemic or circulatory effect. Previous studies have also shown that lesion of nociceptive nerves supplying either the contralateral or the ipsilateral limb prior to inflammatory insults, by using surgery, capsaicin, or local anaesthesia, abolished the contralateral responses [10]. Furthermore, ligating the draining venous system of the inflamed area prior to an insult does not abolish the contralateral response [60]. It is either not likely that the contralateral changes relate to an overcompensation of the non-exercised leg due to disability in the exercised leg. We could thus not observe that the animals limped or showed amended movements or changed behaviors in-between the experimental periods. The abnormalities in the contralateral muscles are also in our opinion too advanced to simply being explained by such a mechanism. Thus, although there are several candidate mechanisms that could cause cross-transfer effects, our findings together with previous well-documented evidence of contralateral effects mediated by the nervous system, suggests that the bilateral effect after unilateral EMS/E is mainly related to neural cross-over signaling mechanisms [1], [2], [61]. A limitation of the study was that other contralateral muscles than the triceps surae muscle were not investigated. In future studies, other contralateral muscles than the homologous muscle should be analyzed.

### Effects of the Electrical Stimulation

It cannot at present be determined to what extent the histological abnormalities in the muscles are a consequence to the use of EMS. It is well known that EMS can have positive effects on muscle regeneration and muscle growth [62], but it is also documented that the effect of EMS differs from voluntary exercise and that it can be deleterious for muscles [27]. Thus, EMS-evoked exercise results in significantly higher degree of muscle injury compared with voluntary exercise [25], [63]. Compared with voluntary contraction, EMS can cause higher force due to an instantaneous recruitment of a very high number of motor-units and a considerably more marked total work performed over time. Furthermore, EMS not only causes contractions but it also depolarizes sensory neurons leading to a large volley to the central nervous system. Thus, contractions produced by EMS can be generated by a combination of recruitment by the stimulating electrodes and central recruitment by the evoked sensory volley [64]. This orthodromic activation of afferent nerve fibers in response to EMS has been shown to cause an induction of interstitial cell proliferation in both stimulated and non-stimulated contralateral muscles [65]. It is therefore possible that some of the observed changes might derive as a consequence of the use of EMS. We have also to keep in mind that EMS might stimulate other structures like contractile components in vessel walls.

### Conclusion

In conclusion, the present study shows that the repetitive unilateral EMS/E used in this study overtime leads to focal muscle tissue changes in form of muscle fiber affection, including fiber degeneration, fiber regeneration and myositis in both the exercised and the non-exercised sides. Furthermore, we also show that there are bilateral changes in the nerve fascicles and in the reactivity pattern of AChE on fibers. These results extend existing evidence of cross-transfer effects after manipulations of one of the extremities and show that unilateral overuse by using EMS/E can have deleterious effects also on the homologous contralateral muscles. Although the exact mechanisms to the morphological contralateral changes observed in this study are unclear, our results indicate that the cross-transfer effects are mediated by the nervous system. Even if these results may not be directly transferable to the human situation, the findings may be important to consider in a wide range of musculoskeletal and neuromuscular disorders. It is also important to keep this effect in mind when using the contralateral muscle as a control muscle in unilateral exercise experiments.
